# Steep switching devices for low power applications: negative differential capacitance/resistance field effect transistors

**DOI:** 10.1186/s40580-018-0135-4

**Published:** 2018-01-28

**Authors:** Eunah Ko, Jaemin Shin, Changhwan Shin

**Affiliations:** 1 Department of Electrical and Computer Engineering, University of Seoul, Seoul, 02504 South Korea; 2 SK Hynix, 2091, Gyeongchung-daero, Bubal-eup, Icheon-si, Gyeonggi-do South Korea

**Keywords:** Steep switching device, Negative capacitance, Phase FET, Low power application, Field effect transistor

## Abstract

Simply including either single ferroelectric oxide layer or threshold selector, we can make conventional field effect transistor to have super steep switching characteristic, i.e., sub-60-mV/decade of subthreshold slope. One of the representative is negative capacitance FET (NCFET), in which a ferroelectric layer is added within its gate stack. The other is phase FET (i.e., negative resistance FET), in which a threshold selector is added to an electrode (e.g., source or drain) of conventional field effect transistor. Although the concept of the aforementioned two devices was presented more or less recently, numerous studies have been published. In this review paper, by reviewing the published studies over the last decade, we shall de-brief and discuss the history and the future perspectives of NCFET/phase FET, respectively. The background, experimental investigation, and future direction for developing the aforementioned two representative steep switching devices (i.e., NCFET and phase FET/negative resistance FET) are to be discussed in detail.

## Introduction

In order to implement better speed/power consumption and integration density (of course, for given cost) in integrated circuit (IC) chip, metal oxide semiconductor field effect transistor (MOSFET) has been scaled down with the help of various process/device solutions over the last a few decades; in fact, the Moore’s Law [1] has been alive for long time. Three-dimensional (3D) device structure, i.e., fin-shaped FET (FinFET), has been adopted for implementing 10 nm-scale technology platform in industry [2]. Although the scaling of semiconductor device has been successfully achieved, the electric field intensity in device (e.g., in the channel region of device) has been hardly controlled (i.e., the electric field has not been constant but increasing every new technology generation). Thereby, the miniaturization of device such as MOSFET and FinFET has evenetually soared up the power density per unit area of IC chip. As of now, many groups have put an emphasis on addressing the technical issue (i.e., the power crisis in IC chip). The root-cause of the ever-increasing power density comes from the fact that threshold voltage (V_th_) has not been appropriately scaled down as much as the physical size of device has been scaled [In the end, power supply voltage (V_DD_) in IC chip has not been scaled down as proportional to the integration density of IC chip]. This is primarily because thermionic emission process (i.e., the physical mechanism of how current in MOSFET forms and flows) causes the fundamental limit of subthreshold slope (SS), which is defined as the gate-to-source voltage (V_GS_) to increase the drain-to-source current (I_DS_) by a factor of 10 in the unit of mV/decade. Note that the value of SS at room temperature is, at best, 60 mV/decade.

In order to overcome the aforementioned bottleneck and to find the alternatives for sub-5-nm technology nodes, various material and/or device solutions have been sought. Among many new exploratory proposals, steep switching device (i.e., transistor/device which has the feature of sub-60-mV/decade SS) is one of the promising suggestions. For example, using the band-to-band tunneling mechanism (instead of the thermionic emission process), tunnel FET (TFET) [3, 4] can achieve sub-60-mV/decade SS. Unfortunately, TFET has more or less “low” on-state drive current as well as the layout penalty due to its “p-i-n” device structure, so that it should be used for a certain specialized application [not as the alternative for conventional complementary metal oxide semiconductor (CMOS) logic technology platform]. Nanoelectromechanical relay (NEM relay) [5] has taken advantages of using the mechanical connection between the channel and the source/drain, to achieve sub-60-mV/decade SS. Although NEM relay has “almost zero” off-state leakage current, it has more or less “poor” endurance characteristics as well as its intrinsic switching time issue (because of its mechanical movement).

In this review paper, we are going to discuss the conspicuous features of the CMOS-compatible steep switching devices for low-power applications; the first one is the negative capacitance field effect transistor (NCFET), and the second one is the phase transition field effect transistor (phase FET). NCFET takes advantages of using the “negative (differential) capacitance” effect of single ferroelectric layer, to achieve the sub-60 mV/decade SS. On the other hand, phase FET uses the “negative (differential) resistance” effect of threshold selectors for implementing the steep switching characteristic. The operational principle, recent results, and challenges of those two steep switching devices are to be shown and discussed.

## Negative (differential) capacitance field effect transistor (NCFET)

Among a few steep switching devices for replacing and/or upgrading the state-of-the-art transistors, the negative (differential) capacitance field effect transistor (NCFET) has received lots of attention [e.g., Two sessions in International Electron Devices Meeting (IEDM) 2017, only for NCFET]. The understanding of how NCFET works, in fact, begins with a simple equation, i.e., the equation for subthreshold slope (SS), which consists of capacitance components in device, as below:

$${\text{SS}} = \frac{kT}{q}\ln \,10 \times \frac{{\partial V_{G} }}{{\partial \varphi_{S} }}$$

$$\frac{{\partial V_{G} }}{{\partial \varphi_{S} }} = m = 1 + \frac{{C_{S} }}{{C_{ins} }},$$

where C_S_ and C_ins_ is the capacitance of semiconductor bulk region and the capacitance of the insulation layer in gate stack, respectively. *φ*_*S*_ indicates the channel surface potential of MOSFET. In the equations above, the “m” factor (which was conventionally thought as being impossible to have the value of “m” factor below “1”) can be lower than “1” by the negative capacitance effect. This would be the main characteristic of NCFET. Because of its advantageous properties such as CMOS process compatibility and scalability, numerous works for developing NCFET are underway, as of 2017; (i) Experimental verification of negative capacitance effect itself, (ii) the steep switching feature of NCFET, and (iii) future research and development of NCFET are going to be discussed in this review paper.

### The debut of negative capacitance field effect transistor (NCFET)

**Fig. 1:**
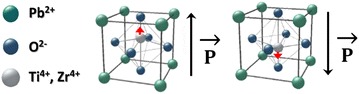
Atomic structure of typical ferroelectric material, Pb(Zr_x_Ti_1−x_)O_3_ (PZT). The atomic structure of ferroelectric material (herein, PZT) is shown. The polarization direction is set by externally applied bias. When the externally applied electric field is stronger than the coercive electric field of the ferroelectric material, the atom at the center of unit cell can move upward or downward, resulting in the switching of polarization state

**Fig. 2:**
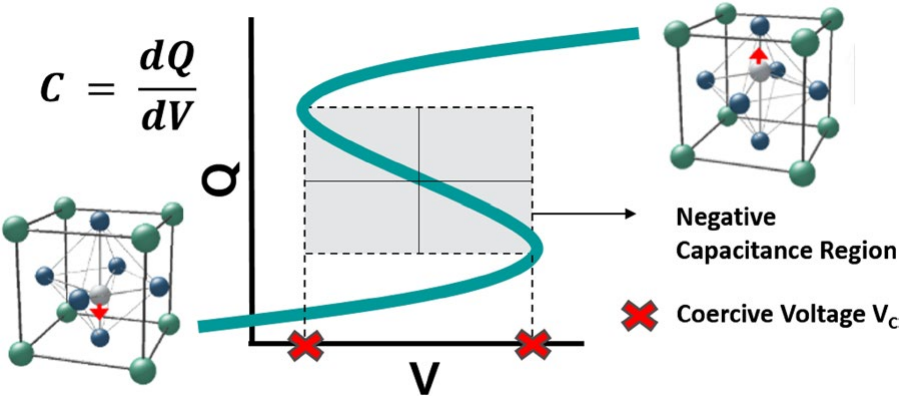
Charge (Q) vs. voltage (V) characteristic of ferroelectric capacitor. The capacitance is defined as the variation of charge over the variation of applied voltage. The red-colored marks represent the coercive voltages of ferroelectric layer. As the externally applied bias becomes higher than the coercive voltages for forward/reverse voltage sweep, the polarization switching occurs. This renders sudden charge reduction in ferroelectric capacitor. This is illustrated as the “S-shaped” curve in the Q vs. V plot

The study of negative capacitance field effect transistor (NCFET) started in earnest at 2008, when S. Salahuddin published the article entitled as “Use of negative capacitance to provide voltage amplification for low power nanoscale devices” in ACS Nano Letters [6]. The letter proposed that conventional metal oxide semiconductor field effect transistor (MOSFET) can overcome its fundamental limit of subthreshold slope, by simply replacing the gate oxidation layer with a well-known ferroelectric material in gate stack (later on, it is known, in many recent works, that the deposition of a ferroelectric material onto the gate oxidation layer is necessary to stabilize the intrinsically-unstable negative capacitance of ferroelectric material). Figure 1 illustrates the atomic structure of a typical ferroelectric material, e.g., Pb(Zr_x_Ti_1−x_)O_3_ (PZT). Essentially, ferroelectric material has two stable states (in terms of its energy), i.e., two polarization states. The polarization state can be altered into the other state, with the help of an externally applied bias (see Fig. 2). To figure out the negative capacitance, let’s suppose that an external voltage is applied to the ferroelectric capacitor (notice that its polarization state was set being aligned in one direction). When the voltage becomes higher than the coercive voltage of the ferroelectric material, the polarization state should be switched in the other state. This would make the sensitivity of charge variation to voltage variation to be negative (i.e., dQ/dV < 0). Since the capacitance is defined as the physical quantity of d*Q*/d*V*, the negative value of dQ/dV (i.e., the negative slope in *Q* vs. *V* plot) indicates the negative differential capacitance. Hence, if we can take the negative value of *C*_*ins*_ in the events of polarization switching, MOSFET can have sub-60-mV/decade SS (see the equation for SS; if C_*ins*_ is negative, the *m* factor is less than 1, resulting in SS < 60 mV/decade at 300 K). In other words, during the polarization switching event of ferroelectric layer, the NCFET (in which a ferroelectric layer is inserted in the gate stack) can have sub-60 mV/decade SS [6–10].

### Experimental demonstration of negative capacitance effect

After the concept of NCFET was suggested, a few pioneering studies have been done to experimentally identify the presence of negative capacitance. Khan et al. have directly measured the negative capacitance, by using a simple RC circuit [11]. The transient response of the RC circuit [in which a 60 nm-thick ferroelectric (PZT) capacitor is connected in series with a resistor] has clearly showed the negative slope in the measured voltage vs. time plot. This should verify the existence and observation of the negative differential capacitance. Appleby et al. have experimentally demonstrated the capacitance of heterostructure capacitor [which is composed of SrTiO_3_ and BaTiO_3_ (BTO) materials], showing that the capacitance increases as a function of BTO thickness; this indicates that the negative capacitance of BTO would contribute to increase the total capacitance [12, 13] (note that the total equivalent capacitance of two series-connected capacitors is lower than the capacitance of any stand-alone capacitor itself). Gao et al. have fabricated “ferroelectric Ba_0.8_Sr_0.2_TiO_3_ + dielectric LaAlO_3_ (LAO)” superlattice heterostructure, which resulted in a higher capacitance than the capacitance of LAO capacitor itself [14]. Similarly, Ku et al. [15] and Sharma et al. [16] have also veryfied the negative capacitance effect of P(VDF_0.75_–TrFE_0.25_) and Hf_0.5_Zr_0.5_O_2_ (HZO) ferroelectric material, respectively.

### Negative capacitance field effect transistor: Saviour of sub-10-nm MOSFET

Since the theoretical proposal of negative capacitance field effect transistor (NCFET) and the direct observation of negative capacitance were published, a clear manifestation of steep switching feature of NCFET has been necessary. In 2015, Jo et al. has done the proof-of-concept study, and they demonstrated the sub-60-mV/decade characteristic of NCFET by conceptually and simply connecting MOSFET in series with ferroelectric capacitor [17, 18]. In [17, 18], it is discussed that the negative capacitance effect enables to amplify the surface potential in baseline device, and thereby, the slope of I_D_–V_G_ curve in sub-threshold region becomes steeper (i.e., subthreshold slope ~ 18 mV/decade at 300 K). Furthermore, Khan et al. demonstrated the short-channel negative capacitance FinFET, with BiFeO_3_ (BFO) ferroelectric capacitor externally connected to baseline device with channel length of 100 nm [19]. Afterwards, many articles which deal with negative capacitance MOSFET [20–24] and FinFET [25–27] demonstrated the reproducible NC effect. Based on state-of-the-art devices such as (i) 2-Dimensional field effect transistors [28–31], (ii) nanoelectromechanical switch [32, 33], (iii) carbon nanotube [34], (iv) silicon-on-insulator (SOI) device [35], and (v) polymer ferroelectric FET [36], recent papers has experimentally and theoretically shown the benefits of using negative capacitance. The number of published studies on NCFET has sharply risen since early 2010s, indicating the increasing interest of NCFET in electron device community (especially, silicon CMOS device community). In next sub-chapters, a few major issues in NCFET design are to be addressed.

#### How to remove “hysteresis” in NCFET?

**Fig. 3:**
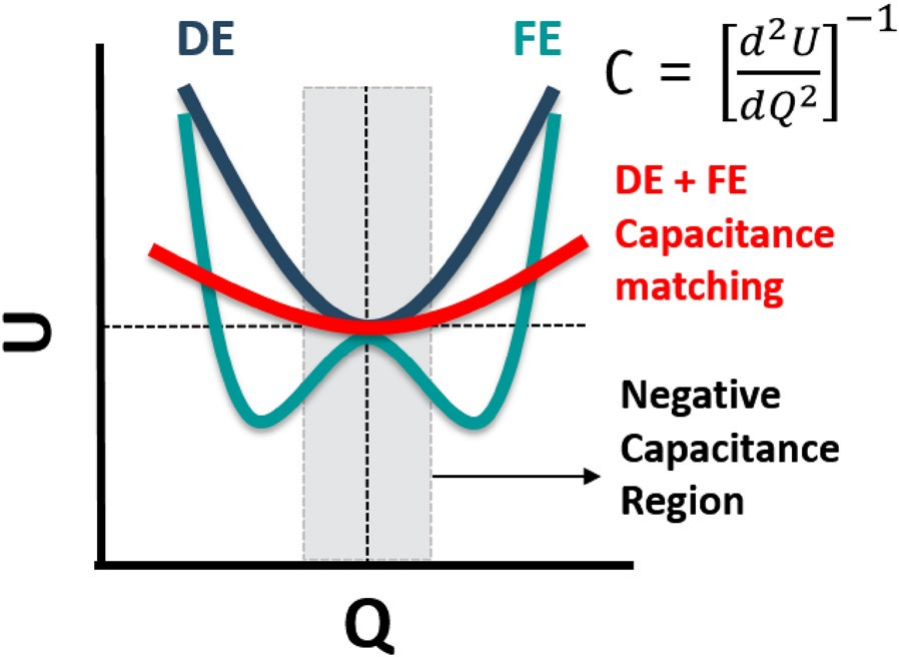
Energy (U) vs. charge (Q) characteristics of ferroelectric capacitor. The navy, green, and red-colored curve represents the U vs. Q curve of dielectric capacitor, ferroelectric capacitor, and capacitance-matched capacitor (i.e., the capacitor which is composed as the series connection of dielectric and ferroelectric capacitor), respectively. By capacitance-matching, the total U vs. Q curve has single energy minimum region, and thereby the hysteresis of ferroelectric capacitor no longer exists

It is indispensable for logic transistors to have the identical threshold voltage for both turn-on and turn-off operation. However, ferroelectric materials used in NCFET intrinsically have their own “hysteresis” characteristic, which hinders the use of NCFETs as logic transistors [6]. Figure 3 shows the energy (U) vs. charge (Q) plots of three difference cases: (i) dielectric capacitor, (ii) ferroelectric capacitor, and (iii) the “dielectric + ferroelectric” capacitor (which is composed as the series connection of the ferroelectric and dielectric capacitor). When two capacitors are connected in series, the red-colored energy vs. charge curve can be introduced (see Fig. 3), which is said to be the capacitance-matching state of the total capacitor system showing one energy minimum point. Thus, the capacitance-matched NCFET can work without any hysteresis (i.e., hysteresis window ~ 0 V). In order to implement the aforementioned stable operation of NCFET by capacitance-matching, there exists two important rules to satisfy modular conditions: (1) the thickness of ferroelectric layer must be decreased until the stable condition is satisfied. This is in line with the continuous scaling of semiconductor devices. In the near future, it is necessary to design the optimal ferroelectric capacitor in terms of the CMOS-compatible material and/or its thickness, and thereafter, the sub-60-mV/decade steep switching and hysteresis-free NCFET can be implemented. (2) The capacitance of baseline device can be appropriately controlled for hysteresis-free operation [20, 26, 37]. In this case, the device parameters that can be adjusted would be varied depending on the device architecture of baseline device. Since there is a trade-off between the subthreshold slope and hysteresis window [1], it should be taken into account that the reduction of hysteresis window would degrade the device performance. For providing the readers with the specific measurement results of  hysteresis, the values of hysteresis from the recent experimental works on NCFET are summarized in Table 1.

#### CMOS-compatibility and scaling

In the early days of NCFET design, people has used typical ferroelectric materials, such as PZT, BFO, and PVDF [11–17]. There are a few reasons to use them: (i) it was easy to fabricate ferroelectric capacitors with these materials. (ii) The polarization switching of typical ferroelectric materials has supremacy, to induce the negative capacitance effect. Yet, those materials had to be fabricated to have the thickness of a few tens of nanometers or even more, so as to have excellent ferroelectric properties. Furthermore, those materials were not compatible to current CMOS process [e.g., a few tens of nanometers ferroelectric layer is too thick to be deposited/included in the current replacement gate (RMG) stack]. To address those technical issues, the hafnium-based ferroelectric material are actively under development. Since the work of fabricating a hafnium-based ferroelectric layer has been published in 2011 [38], many studies have showed that ferroelectric properties can be implemented without doping [39] or with doping various materials (materials such as Y, Al, Zr, Si, etc.) into the hafnium oxide [40, 41]. Lee et al. have announced that it was possible to verify the steep switching characteristic of NCFET with 1 nm-thick HfZrO_x_ ferroelectric layer [42]. Follow-up studies have showed that, by integrating the hafnium-oxide ferroelectric layer (note that the current CMOS process uses the hafnium-oxide as the gate insulation material in mass production), it is possible to improve the CMOS-compatibility of NCFET [43–45]. Moreover, the hafnium-oxide ferroelectric layer can be thin enough for aggressively-scaled transistors (specifically, sub-3-nm-thick hafnium-oxide has been demonstrated!). The last point in this sub-chapter is the effect of domains in ferroelectric layer [46–51]. In developing NCFETs, the domain structure of newly studied hafnium-based ferroelectric should be discussed and scrutinized for accurate device operation.

#### Discussion on internal metal layer

Previous studies which have worked on demonstrating the steep switching feature of NCFET have used an external connection of ferroelectric capacitor to baseline device. To be adopted in aggressively-scaled transistors, the ferroelectric layer should be eventually integrated in the gate stack of baseline device. Numerous studies have reported the performance of NCFET with “integrated” ferroelectric layer. The ferroelectric layer can be fabricated/deposited (i) directly on the dielectric layer or (ii) on top of the internal metal layer (which is deposited on the dielectric layer or interlayer). These two different gate stack structures would have an impact on device reliability. Khan et al. discussed the reliability issues with the aforementioned two different gate stack structures [52]. Depending on the presence of internal metal layer, the conditions required for satisfying the stable operation of device as well as the degree to which the leakage current affects are determined. In this regard, more studies on various issues such as device design, defects, device structural variations, etc., should be done in the near future.

### Future outlook

As mentioned before, the exploratory study on NCFET has begun in 2008. Afterwards, many works have been followed, such as (i) the verification work of negative capacitance phenomenon and the steep switching effect [11–18], (ii) the engineering work to control the hysteresis window [20, 26, 37], (iii) the work for compatibility to current CMOS processes [38–45], (iv) experimental and theoretical studies of capacitance-matching conditions required for stable and reliable operation [53, 54], and (v) modelling for circuit designs [55–60]. Many studies have supported how to adopt NCFET as future ultra-low power logic transistor. However, there are many issues to be tackled, for commercialization. For instance, (1) the recipe of hafnium-based 1 nm-thick ferroelectric layer (especially, with reasonable controllability of defects and thickness), (2) the thorough discussion on the process-induced systematic and random variation of NCFET [61], (3) the discussion on optimal device structure when using NC, (4) the step-by-step capacitance matching manual in wafer-level process, (5) experimentally-verified compact model for NCFET, and (6) AC response and NCFET-based circuit designs [62–64] should be studied in the near future.

## Negative differential resistance FET (Phase FET)

Since the era of “physical” scaling has almost come to an end, people have delved into ways for implementing “energy-efficiency” scaling. Other than using the negative differential capacitance for sub-60-mV/decade MOSFET, the other ways for achieving the sub-60-mV/decade feature have been also investigated. Taking advantages of using resistive switching devices (e.g., Mott insulator), the concept of negative differential resistance FET (a.k.a., phase FET) is proposed. Phase FET is a new class of steep switching devices, and it uses “negative differential resistance” effect (which originates from volatile resistive switching) in threshold selector, which is connected in series to baseline device. Although the history of phase FET is relatively shorter than the other steep switching devices such as Tunnel FET, Impact Ionization MOSFET, etc., it has attracted attentions because of the superior reduction of off-state leakage current and its extremely steep switching characteristic. Various materials such as VO_2_ and Pb(Zr_0.52_Ti_0.48_)O_3_ (PZT) have been adopted for implementing phase FET. In this sub-chapter, the operational principles, history, and recent results of phase FET is to be reviewed and discussed.

**Table 1:** Overview of experimental results of negative capacitance field effect transistors

Refs. no.	Baseline device	Internal metal layer	Ferroelectric material	Minimum subthreshold slope (mV/decade)	Purpose
[17]	MOSFET (Lg = 1 μm)	Yes	P(VDF_0.75_–TrFE_0.25_)	18	First experimental demonstration of steep switching feature
[19]	FinFET (Lg = 100 nm)	Yes	BiFeO3	8.5	Experimental demonstration of negative capacitance FinFET
[20]	MOSFET (Lg = 1 μm)	Yes	P(VDF_0.75_–TrFE_0.25_)	45	Hysteresis-free negative capacitance FET by controlling the drain voltage
[25]	FinFET (Lg = 30 nm)	Yes	HfZrO_2_	55	Negative capacitance FinFET with integrated 5 nm thick hafnium-based ferroelectric layer
[22]	MOSFET (Lg = 10 μm)	No	PbZr_0:52_Ti_0:48_O_3_	13	Integration of 100 nm-thick PZT ferroelectric layer in MOSFET
[26]	FinFET (Lg = 70 nm)	Yes	Pb(Zr_0.2_Ti_0.8_)O_3_	6.8	Hyeresis reduction by adjusting the FinFET’s layout parameter
[42]	MOSFET (Lg = 30 μm)	No	HfZrOx	40.8	Integration of 1.5 nm-thick HZO ferroelectric layer in MOSFET
[27]	FinFET (Lg = 14 nm)	No	Si:HfO	54	Doped Hf ferroelectric material with 3 ~ 8 nm thickness, which is integrated in FinFET
[30]	MoS_2_ 2D FET (Lg = 1 μm)	Yes	HfZrO_2_	6.07	2D negative capacitance FET with integrated hafnia ferroelectric layer

### The debut of negative differential resistance FET (or Phase FET)

There exists materials of which resistivity is varied by external stimulus. For example, Morin. [65] has demonstrated that the resistivity of VO_2_, one of the resistive switching materials, can be significantly modified at a critical temperature. Afterwards, many people have confirmed that the resistivity of VO_2_ can be engineered by (i) carrier concentration, (ii) electrical stimulus (bias), (iii) illumination, (iv) etc. [66–71]. The characteristics of many resistive switching materials have been still under study [72–79]. Thanks to using the resistive switching materials, novel transistors have been invented. In 1988, D. M. Newns et al. proposed a new type of field effect transistor using a Mott insulator (which is used in the channel region) [80]. The Mott insulator channel is directly turned “ON”, by gate bias (i.e., the carrier concentration in channel is significantly increased by gate bias). However, more or less “high” gate voltage is necessary to sufficiently accumulate the carrier concentration for turning “ON” the channel. Instead of using the Mott insulation material, transistors with ionic liquid gate dielectric material have been investigated [81, 82]. It is known that ionic liquid (vs. solid-state-gate-dielectric material) helps to accumulate more carriers to turn “ON” the Mott insulator channel. However, the ionic liquid is susceptible to electrochemical reactions, and it has slow response characteristic. In 2012, Zhou et al. invenstigated the relaxation dynamics of ionic liquid with VO_2_ [83].

**Fig. 4:**
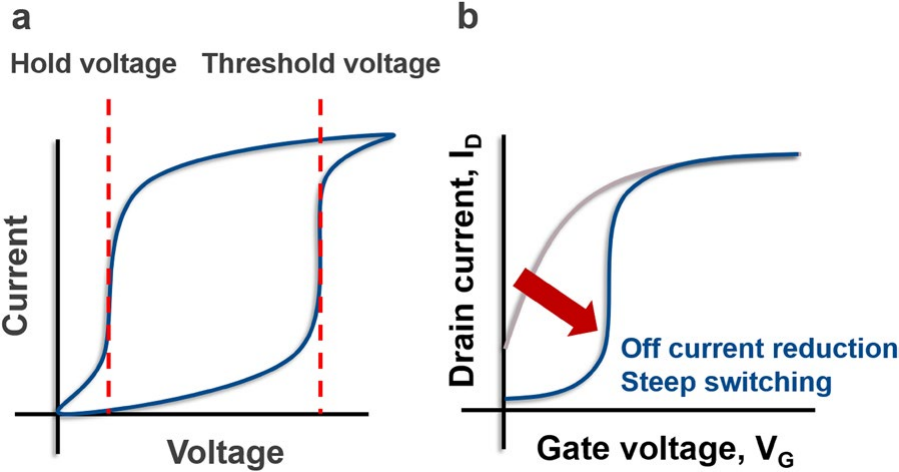
Current–voltage characteristics of TS device and phase FET. **a** Current–voltage (I–V) characteristic of TS device. The TS device is turned on at threshold voltage, and tuned off at hold voltage. **b** Drain current vs. gate voltage (I_D_–V_G_) characteristic of phase FET. Both the suppression of off-state leakage current and steep switching characteristic can be achieved, simply by connecting the TS device in series to baseline transistor

In 2015, Shukla et al. proposed the novel concept of phase FET, for the first time. The phase FET utilized VO_2_ as a threshold selector (TS) [84]. The phase FET is designed and implemented, by connecting VO_2_ device [i.e., threshold selector (TS)] in series to the source region of baseline device. When the phase FET is turned off, the channel resistance becomes high, so that the externally applied voltage to the TS is too low to turn it on. This TS device, in fact, reduces effective gate-to-source voltage and drain-to-source voltage of baseline device. As the gate bias is increased, the channel is turned on, and thereby the resistance is decreased. Therefore, the externally applied voltage to the TS device is increased. When the externally applied voltage to the TS device is higher than the threshold voltage (i.e., the minimum voltage to turn on the TS device), the resistance of TS device is abruptly decreased, so that the applied drain voltage is dropped in the channel, again. As the results of the aformentioned processes, the off-state leakage current is decreased, and on-state drive current is kept. Steep switching characteristic can be acquired because of the abrupt switching characteristic of VO_2_. This concept is illustrated in Fig. 4. In this work, it is confirmed that the on/off-current ratio is enhanced by 20 and 60% for n-type and p-type transistor, respectively. After the debut of phase FET, Frougier et al. presented integrated phase FET using VO_2_ [85] in 2016. In the work, using DC sputtering, VO_2_ was deposited on the source region of baseline device. The on/off-current ratio was increased by 36%, and SS was down to 8 mV/decade at 300 K. In 2017, using SPICE, Aziz et al. analyzed the VO_2_-based characteristics for low power device applications [86, 87].

### Phase FETs using various materials

Phase FET with VO_2_ material has been introduced because the material (VO_2_) has stable resistive switching as well as low “ON” resistance. VO-_2_ is transformed to low resistance state at 340 K. This means that the resistive switching cannot be appropriately used for high temperature applications (e.g., CPU is usually running beyond 340 K). Moreover, the resistance of VO_2_ is lower than that of filament-based TS device (which is explained below, to suppress the off-state leakage current of state-of-the-art transistor). For those aformentioned reasons, many new materials have been proposed to address the technical issues.

**Fig. 5:**
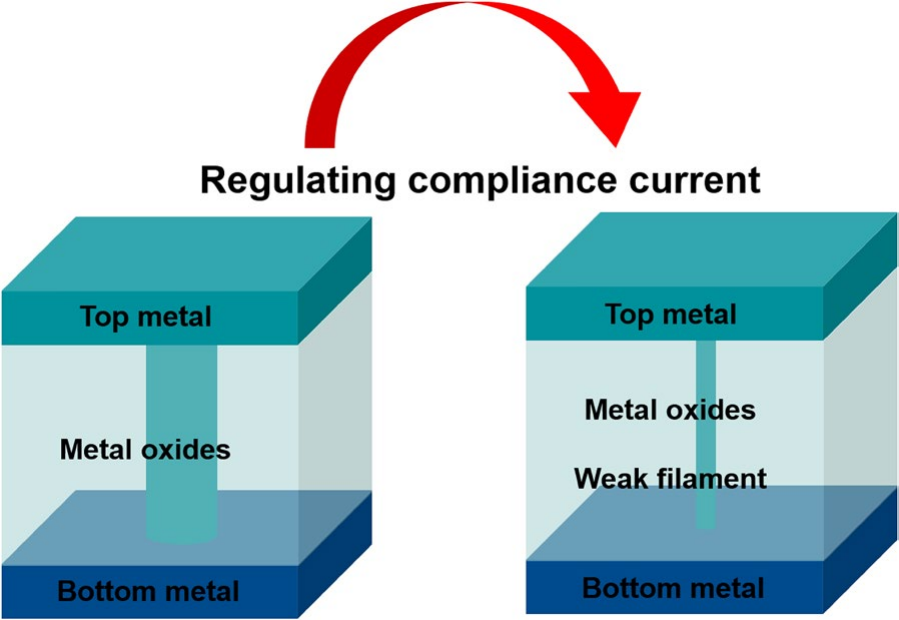
Filament-based threshold selector (TS) device. The Filament of CBRAM can be unstable by modulating compliance current, and therefore, the weak filament is dissolved at hold voltage

Filament-based resistive switching device is originally used for memory device (e.g., CBRAM). However, it was proposed that the CBRAM can be used as the TS device, by simply modulating current flow, i.e., by applying compliance current [88]: by setting low compliance current (specifically, 10–100 μA), the filament can be weakly formed, so that the threshold switching characteristic can be implemented (see Fig. 5). In 2016, Song et al. demonstrated phase FET with TiO_2_-based TS device, for the first time [89]. The TiO_2_-based TS device was turned on at ~ 0.25 V. Moreover, the TiO_2_-based TS has extremely low off-state leakage current characteristic (~ 1 pA). The TiO_2_-based phase FET shows low average SS of < 10 mV/decade at 300 K. However, it was operated as TS device, only under 10 μA compliance current condition. This means that the on-state drive current of the phase FET is very restricted below 10 μA. Moreover, the TS device needs its own delay time (~ 1 μs) to return from low resistance state to high resistance state. In 2016, Lim et al. proposed integrated phase FETs, by connecting CuS_x_-based TS device and “Si–H”-based TS device in series to the drain region of baseline transistor [90]. The threshold selectors (i.e., TS devices) can be turned on at ~ 0.25 V and show ~ 6 orders of abrupt threshold switching at 10 μA compliance current. In 2016, Shukla et al. have also showed the phase FET with HfO_2_-based TS device, for the first time [91]. The HfO_2_-based TS device can be worked as the threshold selector at 100 μA compliance current. They demonstrated ~ 1.5 V of threshold voltage and low off-state leakage current (~ 10 pA). Furthermore, the TS shows the turn-on time of 58 ns and turn-off time of 67 ns. The HfO_2_-based phase FET shows the improvement of on/off-current ratio by ~ 50 × as well as the superior thermal stability (~ 90°C). In 2017, Park et al. suggested NbO_2_-based phase FET, by connecting the TS device in series to the gate electrode of baseline transistor [92]. Although it does not show conspicuous improvement of on/off-current ratio and the TS device used in [92] has relatively high off-state leakage current (~ 1 μA), the steep switching characteristic with such a low off-state leakage current is successfully acquired. In addition to it, the TS needs only ~ 10 ns for recovery (i.e., from low resistance state to high resistance state) and no current flowing limit on current. However, it requires one more resistance to turn-on the TS device, so that there is area penalty issue in layout.

### Future outlook

The phase FET has showed its promising characteristics for low power applications, because of its superior switching feature. However, there is still room for more improvement. The characteristic of filament-based TS device can be determined, most likely by the material, likewise CBRAM. Hence, various metal oxides need to be investigated, to look for best option. For example, ferroelectric tunnel junction (FTJ) [93] can be worked as the TS device in phase FET. Recently, Pb(Zr_0.52_Ti_0.48_)O_3_-based phase FET was introduced by Shin et al. [94].

As described above, for using negative differential resistance effect, the TS device in phase FET can be connected in series to three terminals (i.e., source, drain, and gate terminal) of baseline transistor. The source-connected phase FET (noted as S-phase FET, herein) decreases the effective gate-to-source voltage and drain-to-source voltage. The drain-connected phase FET (noted as D-phase FET, herein) only decreases the drain-to-source voltage. Lastly, the gate-connected phase FET (noted as G-phase FET, herein) only decreases the gate-to-source voltage. The S-phase FET most decreases not only the off-state leakage current but also the on-state drive current. The D-phase FET least decreases the off-state leakage current and the on-state drive current. The G-phase FET shows the intermediate characteristics of both D-phase and S-phase FETs. However, it needs an external resistor. In 2017, Vitale et al. investigated VO_2_-based S- and G-phase TFET [95]. However, VO_2_ cannot show all pros and cons of the proposed device structures, because of relatively high off-state leakage current. Therefore, the best device structure for phase FET should be further scrutinized in the near future.

Finally, the delay time and the on-state drive current of phase FET must be deeply investigated. Various filament-based TS devices have showed more or less slow switching time property (i.e., 0.1–1 μs) as well as low on-state drive current (i.e., 10–100 μA). In 2016, Song et al. showed TiO_2_-based phase FET with AgTe electrode (instead of Ag electrode) [96]. Telluride (Te) helps to dissolve the Ag filament, so that the TS device can be utilized at higher compliance current (~ 100 μA) [97]. Moreover, the delay time is dramatically improved by a factor of ~ 10. Therefore, the method using chalcogenide materials needs to be developed and finely tuned.

## Conclusion

To sum up, we have briefly discussed the concept, recent results, and future outlook for two steep switching devices, i.e., negative differential capacitance FET (a.k.a., NCFET) and negative differential resistance (a.k.a., phase FET). Since 2008, many studies have showed that, by using the effect of negative capacitance on conventional transistors, the switching metric of MOSFET, i.e., subthreshold slope, can become lower than its fundamental limit of 60 mV/decade at 300 K. Furthermore, recent studies have revealed that CMOS-friendly hafnium-based dielectric capacitor can have ferroelectricity, and thereby HfO_2_-based NCFET is currently receiving lots of attentions across countries. The device design for “hysteresis-free”, and the scalability of the ferroelectric layer in the gate stake of MOSFET are the most critical issue to be tackled in the near future, for having current CMOS technology to adopt the NC device. In the middle of 2010s, after the first introduction of resistive switching material-based phase FET, the steep switching characteristic of phase FET was experimentally demonstrated. Because of the simple fabrication process and superiorly low off-state leakage current, various experimental and theoretical studies are ongoing as of early 2018. Although there left many technical issues in material and process, NCFET & phase FET would have lots of potential as a CMOS replacement (or extension) device.
